# Reduction of Aerial Image Misalignment in Face-to-Face 3D Aerial Display

**DOI:** 10.3390/jimaging11050150

**Published:** 2025-05-09

**Authors:** Atsutoshi Kurihara, Yue Bao

**Affiliations:** Graduate School of Integrative Science and Engineering, Tokyo City University, 1-28-1 Tamadutsumi, Setagaya Ward, Tokyo 158-8557, Japan; bao@tcu.ac.jp

**Keywords:** micro mirror array plate, integral photography, aerial image

## Abstract

A Micromirror Array Plate (MMAP) has been proposed as a type of aerial display that allows users to directly touch the floating image. However, the aerial images generated by this optical element have a limited viewing angle, making them difficult to use in face-to-face interactions. Conventional methods enable face-to-face usability by displaying multiple aerial images corresponding to different viewpoints. However, because these images are two-dimensional, they cannot be displayed at the same position due to the inherent characteristics of MMAP. An omnidirectional 3D autostereoscopic aerial display has been developed to address this issue, but it requires multiple expensive and specially shaped MMAPs to generate aerial images. To overcome this limitation, this study proposes a method that combines a single MMAP with integral photography (IP) to produce 3D aerial images with depth while reducing image misalignment. The experimental results demonstrate that the proposed method successfully displays a 3D aerial image using a single MMAP and reduces image misalignment to 1.1 mm.

## 1. Introduction

The technique of displaying images in mid-air has attracted significant attention from researchers and has been the subject of extensive research and development [1,2]. Methods such as Pepper’s Ghost and water vapor-based techniques create the illusion of images floating in the air [2]. The Pepper’s Ghost method primarily consists of a half-mirror and a display serving as a light source. When light emitted from the display passes through a half-mirror tilted at a 45° angle, part of the light is transmitted while the rest is reflected. The reflected light is diffused and, when observed, it appears as a virtual image behind the half-mirror. The water vapor technique, on the other hand, involves creating a screen using water vapor and projecting images onto it. Variants of this method use smoke or microscopic beads as a projection medium [2,3]. In recent years, with the spread of COVID-19, there has been growing interest in contactless aerial displays [4]. Unlike conventional touch panels, aerial displays allow direct interaction with an image projected in mid-air, improving hygiene by reducing physical contact. Various technologies have been developed to form real images in the air, including aerial plasma display systems [5], aerial imaging by retroreflection (AIRR) [6], and transmissive mirror devices (TMDs) [7,8]. The aerial plasma display system generates three-dimensional images by using lasers to ionize air molecules, causing them to emit light. However, due to safety concerns, it is currently recommended that these displays be viewed with protective eyewear. AIRR consists of three main components: a display, a half-mirror, and a retroreflector. It operates as a modified version of the Pepper’s Ghost method. When light from the display reflects off the half-mirror, it is diffused and retroreflected by the retroreflector. A portion of this light then passes through the half-mirror, forming an aerial image. Unlike Pepper’s Ghost, where the virtual image is perceived through human visual processing, AIRR physically converges the light to create an actual aerial image at the intersection of the light source and the half-mirror plane. TMDs are classified into two main types: Dihedral Corner Reflector Arrays (DCRAs) and Micromirror Array Plates (MMAPs). DCRAs [7,8,9,10,11] are optical elements composed of micro-sized corner reflectors arranged on a plate. Some designs feature square holes inside the plate, while others use micro-corner cube reflectors on a base plate. To generate an aerial image, a DCRA requires two key components: the DCRA itself and a display acting as a light source. When viewed along the normal direction of the DCRA, light rays reflected by the corner cubes form an aerial image at the intersection of the light source and the plane of the DCRA. The MMAP [12,13,14,15] used in this study consists of two orthogonal mirror array layers, forming an aerial image in a positional relationship similar to the DCRA. A light ray from the source is transformed into an aerial image when it undergoes an odd number of reflections within the layers. While both DCRAs and MMAPs allow for the easy projection of mid-air images using only a light source, they suffer from limited viewing angles. This limitation prevents conventional aerial displays from being effectively observed in a face-to-face setting [9]. A face-to-face aerial display [16] has been proposed to address this issue, but it results in misalignment when multiple aerial images are displayed. For example, if a user points at an aerial image, the perceived location of the finger varies depending on the viewpoint, causing inconsistencies when multiple users observe the image simultaneously. To overcome this limitation, an omnidirectional 3D autostereoscopic aerial display [4] has been introduced, but it requires multiple expensive and specially designed MMAPs. In this study, we propose a method for displaying aerial images using a single MMAP while reducing image misalignment.

## 2. Conventional Method

### 2.1. The Principle of MMAP

**Figure 1:**
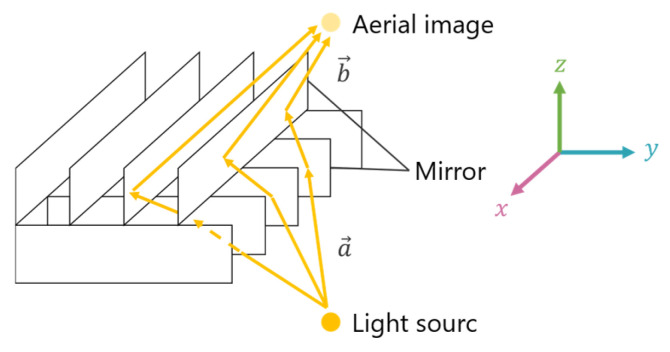
Principle of the MMAP.

Figure 1 illustrates the structure of the MMAP, which consists of two layers of orthogonal mirror arrays.

When light rays from a source are reflected an odd number of times in each layer, an aerial image is formed at a position that is plane-symmetric to the light source. For example, if the xyz coordinate system is aligned with the mirror surfaces of each layer, the incident vector “a” and the ray vector “b”, which are reflected once in each layer, can be expressed by Equations (1) and (2). From these equations, when viewing the MMAP from the z-axis direction, it is evident that the rays forming the aerial image are retroreflected by the mirror array. Additionally, since the z-component of the rays remains unchanged before and after passing through the MMAP, the aerial image appears at a position that is plane-symmetric with respect to the MMAP.

(1) $$\vec{a}=\left(a_{x},a_{y},a_{z}\right)$$

(2) $$\vec{b}=\left({-a}_{x},{-a}_{y},a_{z}\right)$$

Furthermore, as summarized in Table 1, the nature of the image depends on the number of reflections in each MMAP layer [17].

When light rays emitted from a source undergo an odd number of reflections in each layer, an aerial image is formed. However, if light rays are reflected an odd number of times in one layer and an even number of times in the other, the resulting image is a specular reflection, commonly referred to as a “ghost” image. When each layer reflects the light an even number of times, the rays pass directly through the MMAP without forming an image. The number of reflections within the structure primarily depends on the incidence angle of the incoming light.

### 2.2. Integral Photography

**Figure 2:**
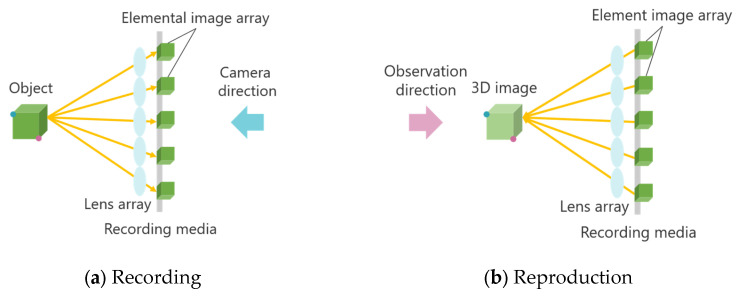
Principle of Integral Photography (IP).

Integral Photography (IP) is a 3D display technology based on light ray reproduction using a lens array [18,19]. This technology is primarily divided into two stages: recording the captured object and reproducing the 3D image. Figure 2 illustrates the principle of the IP method.

First, an object is photographed through a microlens array. The light emitted from the object passes through the microlens array, forming an Elemental Image Array (EIA), which consists of small images called elemental images. The number of elemental images corresponds to the number of lenses in the array. The same lens array is then placed in front of the recording medium on which the EIA is captured. At this stage, light rays from the elemental images pass through each lens, traveling in the opposite direction compared to when the image was originally recorded. As a result, a 3D image is reconstructed, emitting light in a manner similar to that of the original object. A one-dimensional (1D) lens array provides unidirectional disparity, while a two-dimensional (2D) lens array provides both horizontal and vertical disparity. However, in IP, the shooting and viewing directions are opposite, causing the depth of the 3D image to be reversed compared to that of the original object. Figure 2 demonstrates this effect: when the object is observed from the shooting direction, the red point appears in front of the blue point, but when the 3D image is viewed, the blue point appears in front of the red point. To address this depth inversion issue, methods such as rotating each elemental image by 180° [20] and using a concave lens array during recording [21] have been proposed. However, when a 3D image is further re-imaged as a 3D aerial image using MMAP, the depth inversion occurs twice, effectively canceling out the depth reversal and restoring the correct depth perception.

### 2.3. Face-to-Face Aerial Display

**Figure 3:**
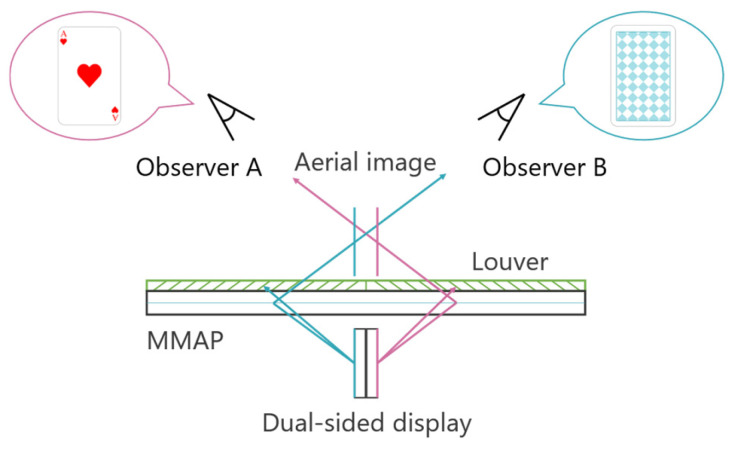
Principle of the face-to-face aerial display.

A technique has been developed that utilizes two displays as light sources, allowing aerial images to be observed from two different directions. Figure 3 illustrates the configuration of this face-to-face aerial display.

The light emitted from the two displays is re-imaged by the MMAP, producing two aerial images that can be viewed from each observer’s position. By ensuring that the images displayed on each screen correspond to the respective viewpoints, different perspectives can be presented. In this case, magenta is the ray and aerial image for observer A, and cyan is the ray and aerial image for observer B. For example, when playing cards are displayed as aerial images, Observer A can see the front side of the cards, while Observer B can see the back side. Additionally, louvers are placed on the MMAP to eliminate light rays from sources that do not contribute to the formation of the aerial images. This prevents the original display images from being visible through the MMAP. However, since the generated aerial images are two-dimensional (2D), there is a separation between them due to the thickness of the display panels. In other words, the aerial images cannot be projected at exactly the same position in space.

### 2.4. Omnidirectional 3D Autostereoscopic Aerial Display

**Figure 4:**
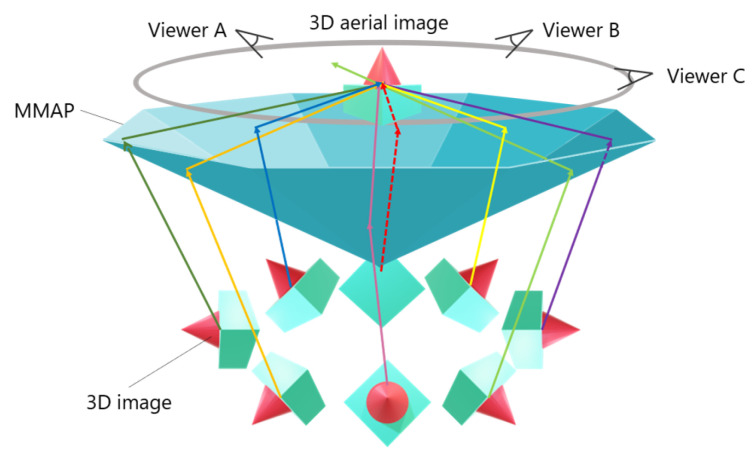
Configuration of the omnidirectional 3D autostereoscopic aerial display.

An omnidirectional 3D autostereoscopic aerial display has been developed as a method for multiple observers to view aerial images from the same location. Figure 4 illustrates the configuration of this aerial display.

**Figure 5:**
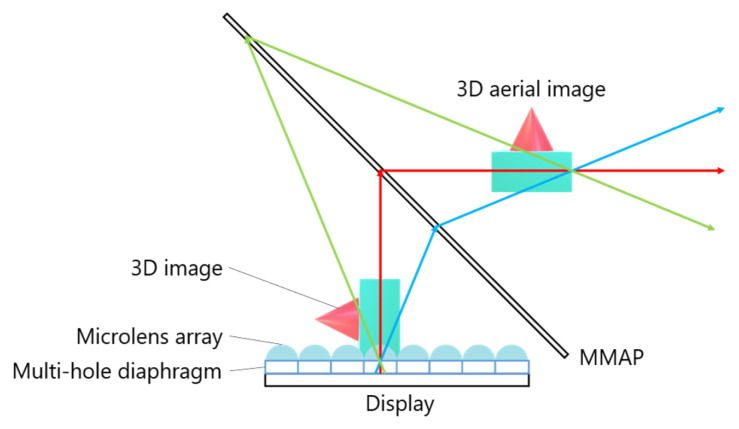
Display procedure of the conventional system.

This system primarily consists of multiple IP display devices and MMAPs. The MMAPs are arranged in an isosceles triangular configuration and combined to form a concave polyhedral shape. By aligning these MMAPs and 3D images at appropriate angles, a 3D aerial image can be displayed, allowing multiple viewers to observe it simultaneously. Figure 5 illustrates the process of generating a 3D aerial image. A 3D image is first created by placing a microlens array (MLA) over a set of elemental images (EIA) displayed on a screen. This 3D image is then re-imaged as a 3D aerial image by propagating through the corresponding MMAP. Multiple IP display devices and their corresponding MMAPs work together to produce a 3D aerial image that is viewable from all directions. Additionally, a multi-hole diaphragm is used to restrict the range of emitted light from the 3D image, effectively reducing ghosting effects. However, a major drawback of this system is the requirement for multiple high-cost and specially shaped MMAPs.

## 3. Proposed Method

**Figure 6:**
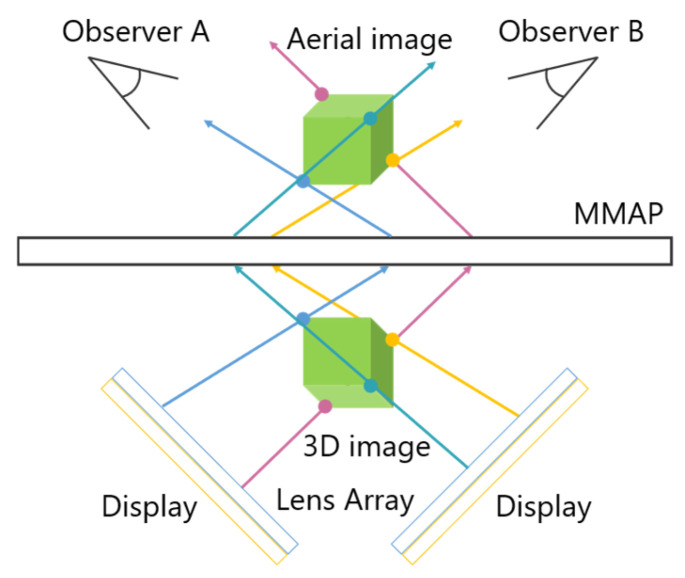
Principle of the proposed method.

Conventional face-to-face aerial displays produce 2D aerial images, resulting in gaps between each image. While omnidirectional 3D aerial displays can eliminate these gaps, they require multiple specially shaped and expensive MMAPs. Therefore, this study proposes an aerial image display method that enables multi-view observation of 3D aerial images at the same location using a single MMAP. In the proposed method, the aerial image displayed in a face-to-face aerial display is converted into a 3D image to introduce depth, and multiple aerial images are superimposed. As in conventional methods, Integral Photography (IP) is used as the 3D display technique. Figure 6 illustrates the principle of the proposed method. Cyan, magenta and yellow are examples of the vertices of the aerial image and the rays that comprise it.

Two displays are used, similar to conventional face-to-face aerial displays. These displays are tilted, with an optimal tilt angle of 45°, as the MMAP produces the most luminous aerial image at a 45° angle relative to the normal direction [4]. The EIA is displayed on the screen, and a corresponding lens array is used to generate 3D images via IP. These 3D images are captured from different viewpoints so that they change based on the observer’s position. Additionally, the imaging position of the 3D images and the arrangement of the equipment are adjusted so that the 3D images overlap. The 3D images displayed in this manner serve as light sources for the MMAP and are re-imaged as aerial images. Since the MMAP forms aerial images at positions that are plane-symmetric to the light source, each aerial image is displayed in the same spatial relationship as the corresponding 3D image. As a result, observers can view the aerial images that are at the same location. This approach is expected to reduce the misalignment between aerial images, a problem commonly encountered in conventional methods. Furthermore, unlike the omnidirectional 3D autostereoscopic aerial display, which requires multiple MMAPs, the proposed method allows multiple aerial images to be displayed using a single MMAP, thereby reducing equipment costs.

**Figure 7:**
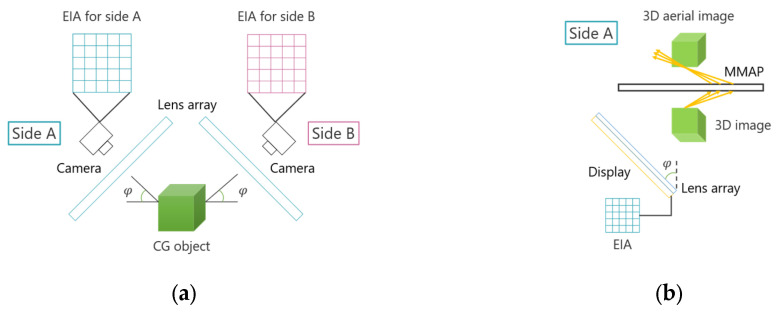
Flow of the proposed method. (**a**) Obtaining EIAs in the CG space. (**b**) Displaying the 3D aerial image in the real space.

Figure 7 illustrates the workflow of the proposed method. Since most of the light rays forming the aerial image travel obliquely relative to the MMAP, it is preferable to observe the image from an oblique angle. Therefore, when capturing CG objects using IP, it is necessary to consider the optimal viewing angle of the aerial image. As shown in Figure 7a, the lens array and camera are tilted at an angle φ relative to the CG object being captured. This setup produces an EIA that represents the subject as seen from an inclined viewpoint. To ensure proper alignment of the 3D images, the positional relationship between the center coordinates of the CG object and each lens array must be adjusted so that they are identical. Next, as illustrated in Figure 7b, a 3D image is generated by displaying the EIA of side A on the screen and placing a lens array with the same specifications as the CG space over it. When light emitted from this 3D image passes through the MMAP, a 3D aerial image of side A is obtained. A similar process is applied to side B, and by appropriately adjusting the positions of each image, aerial images can be displayed that are observable from both sides at the same position.

## 4. Experiment

Two experiments were conducted to verify the effectiveness of the proposed method. The first experiment aimed to evaluate the performance of the proposed method in displaying aerial images. The second experiment measured the misalignment between the two displayed aerial images. Details of the equipment used in these experiments are provided in Table 2. In these experiments, lenticular lenses, which are relatively easy to obtain, were used as the lens arrays. As a result, motion parallax was only present in the horizontal direction relative to the aerial image. Additionally, due to the size limitations of the MMAPs used in this experiment, the light from each display was insufficient, sometimes causing the aerial images to be obscured. To mitigate this issue, the position of the MMAPs was adjusted accordingly.

### 4.1. Photographing the CG Object with Integral Photography (IP)

**Figure 8:**
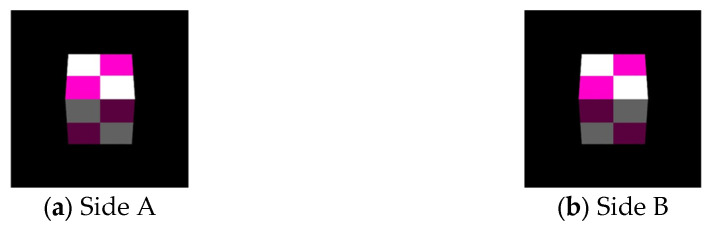
CG Object for capturing.

Since the proposed method displays 3D aerial images instead of 2D, the MMAP light source must also consist of 3D images. Therefore, EIAs are required for each viewpoint to generate 3D images using IP. In this experiment, the target object is first photographed in the CG space using IP. Pov-Ray (version 3.7.0) was used as the 3D CG creation software. Figure 8 shows the CG object used as the subject in this experiment.

**Figure 9:**
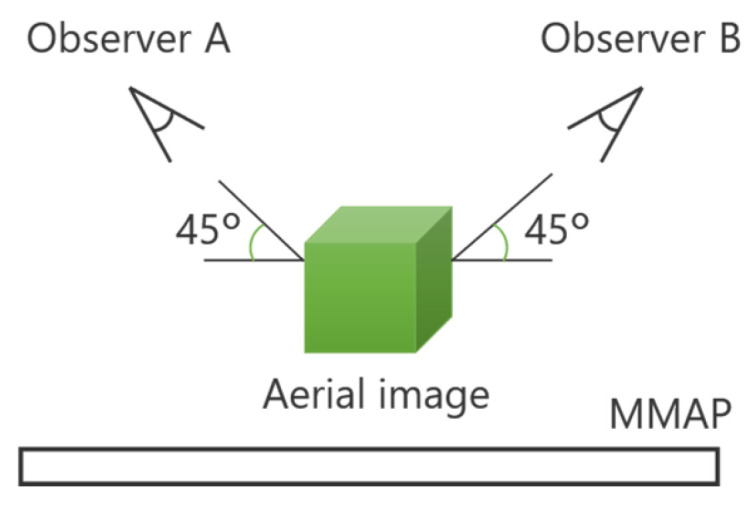
Observation of aerial images in the proposed method.

Because the brightness of the aerial image is maximized when the MMAP is positioned at a 45° angle to the normal direction, the CG object was captured in a way that allowed the 3D aerial image to be observed from a position 45° above the vertical axis, as shown in Figure 9.

**Figure 10:**
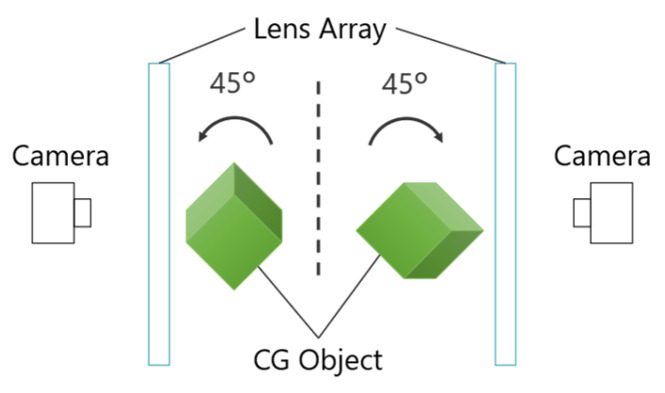
Photographing the CG object.

To simplify the image-capture process, the positions of the camera and lenticular lens were fixed, while the CG object was rotated clockwise and counterclockwise by 45° and captured separately. Figure 10 provides an overview of the image-capture setup for the CG object.

### 4.2. Experiment 1: Evaluation of 3D Aerial Images

**Figure 11:**
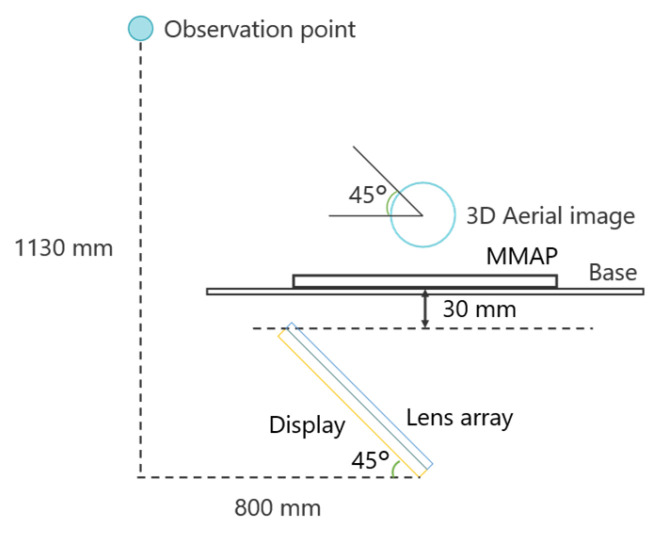
Configuration of the experimental device in experiment 1.

Conventional aerial displays typically show 2D images as aerial images, whereas this research uses IP to display 3D aerial images. To evaluate the performance of the aerial images displayed by the proposed method, 18 men and women in their 20s and 30s were asked to assess the clarity of the aerial images. The configuration of the experimental device used for the evaluation is shown in Figure 11. The subjects’ faces were positioned at the observation point, and they were asked to observe the aerial image from a 45° angle.

Subjects observed the aerial images from a position 45° above the vertical direction, and the evaluation was conducted using Scheffe’s paired comparison, comparing each aerial image displayed at different positions. The evaluation was performed on a 5-point scale: 2, 1, 0, −1, and −2. Scheffe’s paired comparison involves selecting two objects to be evaluated and comparing which one is superior. For example, A’s aerial image is compared with B’s aerial image, and if A is judged to be superior, a positive score is assigned; if B is judged to be superior, a negative score is assigned. This trial was conducted for all combinations in random order. After all comparisons were completed, the scores for each evaluation target were tallied. Since this experiment used Nakaya’s method of Scheffe’s pairwise comparison, which does not consider the order in which the evaluation targets are displayed, the average evaluation value $\hat{\alpha_{i}}$ is obtained from Equation (3) when the total result for all subjects is $x_{i}$, the number of subjects is t, and the number of evaluation targets is n. The evaluation target with a larger average evaluation value is considered superior [22].

(3) $$\hat{\alpha_{i}}=\frac{x_{i}}{tn}$$

**Figure 12:**
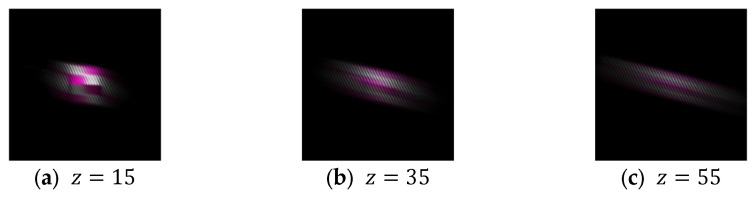
EIA corresponding to each display position of the aerial image.

**Figure 13:**
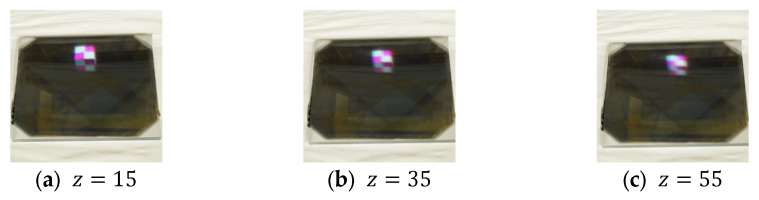
Aerial images at each display position.

**Figure 14:**
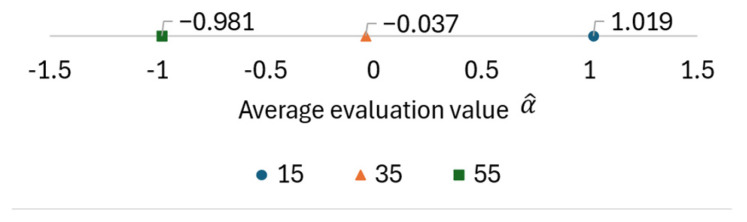
Evaluation results of aerial images by average evaluation value.

The display position of the aerial image is determined by the positional relationship between the lens array and the subject CG object during the IP shooting phase. In this experiment, the center of curvature of the lens array is placed at the origin in the CG space, and the subject is moved in 20-unit increments from the origin in the positive direction along the z-axis. The camera is also moved 100 units in the negative direction along the z-axis so that the subject can be photographed through the lens array. Therefore, the position of the subject is determined by the z-component of the coordinates of the subject’s center, and only this z-component was used to adjust the position of the aerial image. The EIA used to display each aerial image and the resulting displayed aerial images are shown in Figure 12 and Figure 13, respectively. The results of the evaluation of the aerial images by each subject are provided in Table 3, and the average evaluation values for each evaluation target obtained from this data using Equation (3) are shown in Figure 14. The numbers in the columns of Table 3 represent the display position of the aerial images, and these aerial images are presented in order from left to right.

### 4.3. Evaluation 2: Measuring Misalignment of Aerial Images

**Figure 15:**
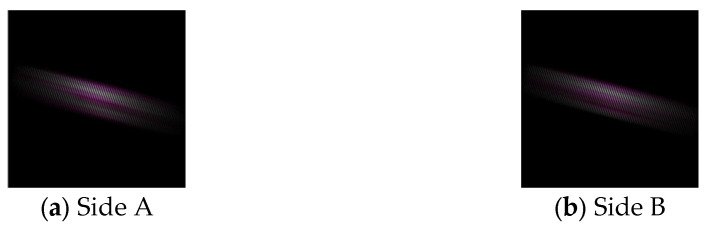
EIA used in the measurement experiment.

**Figure 16:**
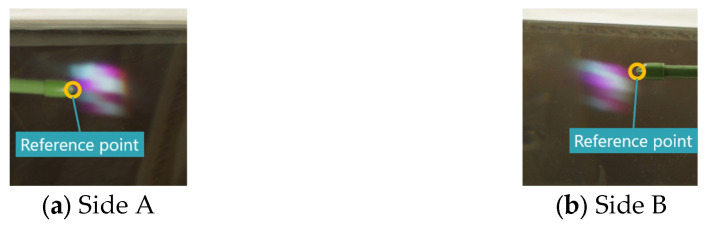
Captured aerial images (proposed method).

**Figure 17:**
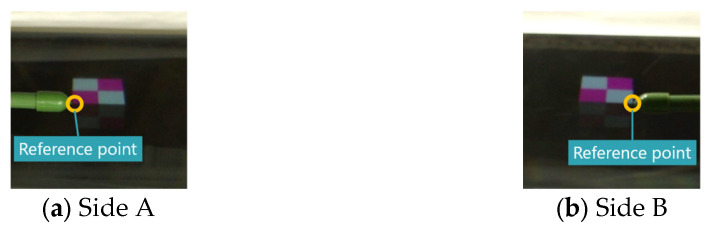
Captured aerial images (conventional method).

We measured the misalignment of the aerial images formed by each display. In the proposed method, the z-coordinate in CG space of the object used for the aerial images is 65. The Elemental Image Array (EIA) used to display the 3D images is shown in Figure 15, and each aerial image captured from a position 45° to the vertical direction is shown in Figure 16. In this experiment, one vertex of the cube was designated as the reference point, which was indicated using the tip of an indicator stick, as shown in Figure 16. Unlike real objects, accurately determining the position of a 3D aerial image is challenging due to the narrow viewing area and limited resolution. Therefore, the camera position was adjusted so that the aerial image was centered within the camera’s field of view when tilted at 45°. The position of the indicator rod was then adjusted while observing the camera feed. For the conventional method, the aerial image was displayed using Figure 8, with the reference point set at the same location as in the proposed method. However, since the aerial images generated by the conventional method are 2D rather than 3D, the reference points of each aerial image are aligned in height, as shown in Figure 17.

**Figure 18:**
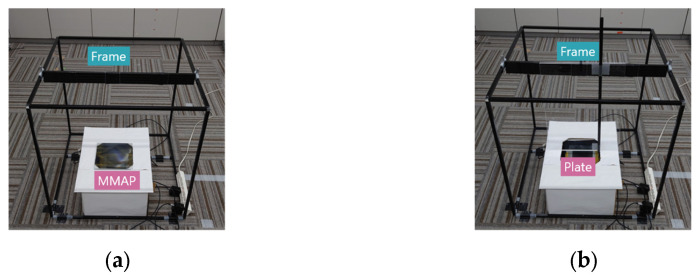
Experimental device in experiment 2. (**a**) Side view (with MMAP, without a plate). (**b**) Top view (without MMAP, with a plate).

**Figure 19:**
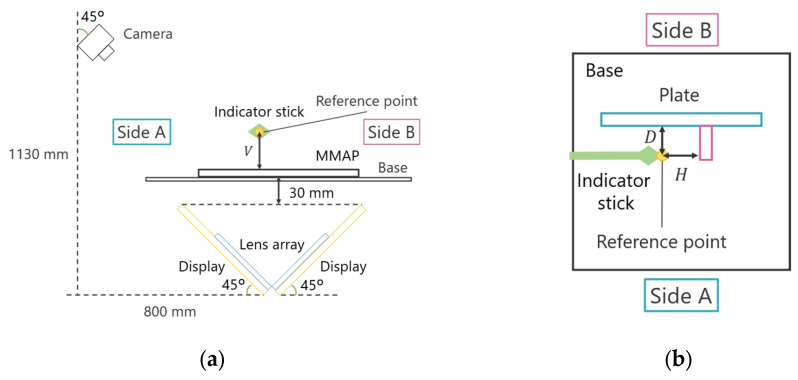
Configuration of the experimental device in experiment 2. (**a**) Side view (with MMAP, without a plate). (**b**) Top view (without MMAP, with a plate).

The position of the support bar pointing to the reference point was measured for each aerial image in the depth, horizontal, and vertical directions, and the difference was recorded as the displacement of the aerial image. To minimize measurement errors, each viewpoint was measured ten times, and the average value was used as the final measurement result. Figure 18 and Figure 19 illustrate the experimental setup and configuration.

The position of the indicator in the depth and horizontal directions is represented by the distances D and H from the reference plate, respectively, while the position in the vertical direction is represented by the height V from the MMAP. For example, when measuring the position of an aerial image on Side A, the height of the indicator rod is measured first. The MMAP is then removed from the experimental setup, and a reference plate is placed within a frame surrounding the experimental apparatus. This plate serves as a reference for measuring the indicator rod’s position in the depth and horizontal directions. The same procedure is applied for measurements on Side B. The measured misalignment results for the aerial images are presented in Table 4 and Table 5, which correspond to the conventional face-to-face aerial display and the proposed method, respectively.

## 5. Discussion

**Figure 20:**
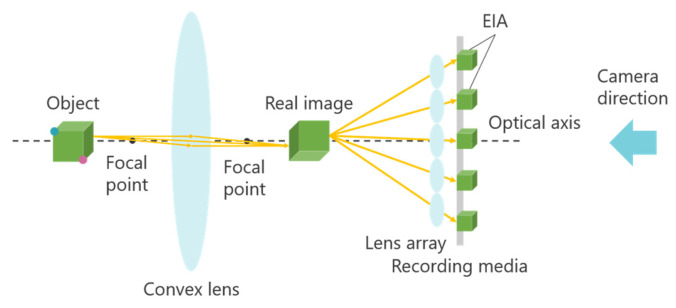
Recording with a convex lens in IP.

**Figure 21:**
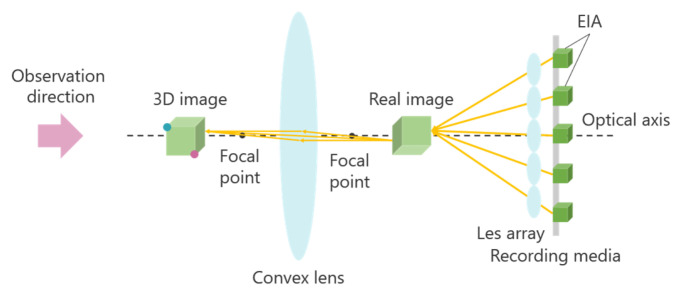
Reconstruction with a convex lens in IP.

In Experiment 1, several subjects evaluated the clarity of the 3D aerial images. Figure 13 indicates that a smaller z-value results in a sharper aerial image, whereas a larger z-value reduces sharpness. This suggests a trade-off between the sharpness of the aerial image and its display position. The deterioration in sharpness as the display position moves farther away is likely due to the increased distance between the subject and the lens array when the CG object is captured in the integral photography (IP) system. IP records the direction and intensity of light by passing it through a lens array. However, as the distance between the subject and the lens array increases, light from the subject becomes more diffused, making recording difficult. This leads to a decrease in the resolution of the 3D image during playback. To mitigate this issue, a method utilizing a convex lens is considered. Figure 20 and Figure 21 illustrate the recording and reconstruction process of an IP system incorporating a convex lens.

First, a convex lens is placed between the object and the lens array. The object is positioned beyond the focal length of the convex lens to form a real image. The lens array then captures this real image to create an elemental image array (EIA). During playback, the same optical arrangement is used to reconstruct the real image from the EIA, and a 3D image of the original object is obtained using a convex lens. By employing this approach, the distance between the lens array and the object can be reduced, while the distance between the lens array and the 3D image increases, effectively extending the display position while maintaining 3D image resolution.

In Experiment 2, the misalignment of two displayed aerial images was measured and compared between the conventional and proposed methods. Table 4 and Table 5 show that the depth misalignment of the aerial image is 20.5 mm for the conventional method and 1.1 mm for the proposed method, demonstrating that the proposed method significantly reduces misalignment. However, the proposed method exhibits errors of 0.4 mm, 1.1 mm, and 1.0 mm in the vertical, depth, and horizontal directions, respectively. To assess measurement accuracy, we first consider measurement errors. In this analysis, the true value is assumed to be zero, and misalignment in each direction is evaluated using a *t*-test. Table 6 and Table 7 present the 95% confidence intervals for aerial image misalignment in each method, along with the parameters used in the *t*-test. Statistically insignificant *p*-values (*p* ≥ 0.05) are highlighted in bold.

For example, the average value of V in the conventional method does not significantly differ from the true value of zero (*p* ≥ 0.05). Similarly, other measurements also show no significant difference compared to the true value of zero. These results indicate that the measurement outcomes are reliable. Therefore, it is inferred that aerial image misalignment can be minimized by appropriately adjusting the distance between the CG object and the lens array when capturing images in the IP system. If misalignment persists, it is believed that adjusting the display position can enable fine-tuning to fit the display environment.

There are several challenges in the practical application of the display device in the proposed method, such as the large size of the device and the luminance of the aerial image. Regarding the former, a larger MMAP is required. Since the image on the display must be smaller than the MMAP and the MMAP itself is expensive, increasing the size of the proposed device is impractical even if technically feasible. Regarding the latter, the luminance of the aerial image must be maintained in the presence of ambient light. This is because the luminance of the aerial image itself is low and is therefore outweighed by the brightness of the ambient light reflected from the top of the MMAP. A simple solution is to use a high-luminance display for the display device; however, such displays are expensive. Therefore, a method in which a polarizing plate and a quarter-wave plate are placed above the MMAP is considered. Ambient light passing through these two optical elements first becomes circularly polarized. When this light is reflected at the top of the MMAP, it passes through the quarter-wave plate and becomes linearly polarized. Since this linearly polarized light oscillates in a direction perpendicular to the direction of the polarizer slit, it is blocked by the polarizer. Therefore, it is believed that the proposed device can be used in environments with ambient light. Considering the above, although increasing the size of the proposed device is challenging, it can be used in locations other than dark environments, and it is considered that the practical application of the proposed device is feasible.

## 6. Conclusions

In this paper, we focused on the problem of aerial image misalignment in conventional face-to-face aerial displays and the number of MMAPs used to display the aerial image. The proposed method was designed to reduce the cost of the equipment while mitigating the misalignment of the aerial images. To verify the effectiveness of the proposed method, we measured the misalignment of the two displayed aerial images and compared the results for each method. Although the positions of the two aerial images displayed using the proposed method contain some errors, the proposed method demonstrated a reduction in misalignment compared to the conventional method. Therefore, it was confirmed that the proposed method can reduce the misalignment of aerial images while decreasing the number of MMAPs required. The proposed method has potential applications in fields such as medical systems, which require non-contact operation and precise pointing.

## Figures and Tables

**Table 1:** Relationship between images and number of reflections by the MMAP.

		First Layer	
		Odd Times	Even Times
Second layer	Odd times	Aerial image	Ghost
	Even times	Ghost	Transmitted light

**Table 2:** Details of the experimental equipment used in each experiment.

Equipment	Parameters	Specification
MMAP	model number	ASKA3D-200NT
	size	200 × 200 mm
	pitch	0.3 mm
	viewing angle	40°
	material	optical resins
Display	model number	JN-MD-IPS133UHDR-T
	body size	312 × 194 × 11 mm
	screen size	13.3 inch
	resolution	3840 × 2160 pixel
Lens array	type	lenticular lens
	LPI	22
	thickness	3.0 mm
	index of refraction	1.49
	material	acrylic
Camera	model number	SONY α6000
	lens	E 30 mm F3.5 Macro
	resolution	6000 × 4000 pixel
	ISO	100
	shutter speed	1/5 s
	f-number	f/3.5
Plate for the measurement experiment	material	acrylic
	index of refraction	1.49
	thickness	10.0 mm

**Table 3:** Results of each subject’s evaluation of the clarity of aerial images.

Display Position (*z*)	15–55	55–35	15–35
Subject 1	2	−1	1
Subject 2	2	−1	1
Subject 3	2	−2	1
Subject 4	2	−1	1
Subject 5	2	−2	2
Subject 6	2	−2	1
Subject 7	2	−2	2
Subject 8	2	−1	1
Subject 9	2	−1	1
Subject 10	2	−1	2
Subject 11	2	0	2
Subject 12	2	−2	2
Subject 13	2	−1	1
Subject 14	2	−1	2
Subject 15	2	−2	2
Subject 16	2	−1	1
Subject 17	2	−2	2
Subject 18	2	−2	1

**Table 4:** Measurement results of aerial image misalignment (conventional method).

	Side A (mm)			Side B (mm)			Misalignment (mm)		
	V	D	H	V	D	H	V	D	H
1st	127.5	18.5	80.3	127.8	17.5	79.5	−0.3	1.0	0.8
2nd	127.8	18.8	80.5	128.0	17.3	79.8	−0.2	1.5	0.7
3rd	127.5	18.5	80.5	128.0	17.5	79.5	−0.5	1.0	1.0
4th	127.3	18.5	80.8	127.5	17.8	79.5	−0.2	0.7	1.3
5th	127.5	18.8	80.5	127.8	17.5	79.8	−0.3	1.3	0.7
6th	127.3	18.5	80.5	128.0	17.5	79.5	−0.7	1.0	1.0
7th	127.8	19.0	80.3	128.3	17.8	79.8	−0.5	1.2	0.5
8th	127.5	18.8	80.8	128.0	17.8	79.5	−0.5	1.0	1.3
9th	127.8	18.8	80.5	127.8	17.5	79.3	0.0	1.3	1.2
10th	127.5	18.8	80.8	127.8	17.8	79.5	−0.3	1.0	1.3
Avg.	127.6	18.7	80.6	127.9	17.6	79.6	−0.4	1.1	1.0

**Table 5:** Measurement results of aerial image misalignment (proposed method).

	Side A (mm)			Side B (mm)			Misalignment (mm)		
	V	D	H	V	D	H	V	D	H
1st	123.3	16.5	75.8	123.5	17.5	76.0	−0.2	−1.0	−0.2
2nd	123.0	16.8	76.0	123.8	17.3	75.5	−0.8	−0.5	0.5
3rd	123.5	17.0	75.8	124.0	17.5	75.8	−0.5	−0.5	0.0
4th	123.8	17.3	75.5	123.8	17.8	75.5	0.0	−0.5	0.0
5th	123.3	16.8	76.0	123.5	17.3	76.0	−0.2	−0.5	0.0
6th	123.8	16.5	75.8	123.8	17.5	75.8	0.0	−1.0	0.0
7th	123.3	16.8	76.0	124.3	17.8	75.8	−1.0	−1.0	0.2
8th	123.5	16.8	75.8	124.0	17.5	75.5	−0.5	−0.7	0.3
9th	123.3	16.5	76.0	123.8	17.5	76.0	−0.5	−1.0	0.0
10th	123.5	17.0	76.0	124.0	17.3	75.5	−0.5	−0.3	0.5
Avg.	123.4	16.8	75.9	123.9	17.5	75.7	−0.4	−0.7	0.1

**Table 6:** Calculation results for average, standard deviation, 95% confidence interval, t-value and *p*-value of the misalignment (conventional method).

		V	D	H
Avg.		0.7	−20.5	−0.9
SD		0.31	0.30	0.34
95% CI	Lower limit	0.51	−20.67	−1.06
	Upper limit	0.89	−20.29	−0.64
t-value		0.07	−1.96	−0.09
*p*-value		0.95	0.08	0.93

**Table 7:** Calculation results for average, standard deviation, 95% confidence interval, t-value, and *p*-value of the misalignment (proposed method).

		V	D	H
Avg.		−0.4	1.1	1.0
SD		0.19	0.21	0.28
95% CI	Lower limit	−0.47	0.97	0.81
	Upper limit	−0.23	1.23	1.15
t-value		−0.02	0.07	0.09
*p*-value		0.98	0.94	0.93

## Data Availability

The authors confirm that the data supporting the findings of this study are available within the article.
